# Morphological Changes of Calcium Carbonate and Mechanical Properties of Samples during Microbially Induced Carbonate Precipitation (MICP)

**DOI:** 10.3390/ma15217754

**Published:** 2022-11-03

**Authors:** Zhaorui Gu, Qing Chen, Lishuang Wang, Shuang Niu, Junjie Zheng, Min Yang, Yunjun Yan

**Affiliations:** 1 Key Laboratory of Molecular Biophysics of the Ministry of Education, College of Life Science and Technology, Huazhong University of Science and Technology, Wuhan 430074, China; 2 Institute of Geotechnical and Underground Engineering, Huazhong University of Science and Technology, Wuhan 430074, China

**Keywords:** calcium carbonate, crystal morphology, MICP, mechanical properties, nucleation sites

## Abstract

Recently, microbially induced carbonate precipitation (MICP) has shown potent potential in the field of civil engineering. The calcium carbonate crystals produced by bacteria during the MICP process play a central role in sticking the soil. However, the morphological changes of calcium carbonate crystals in this process and the mechanical performance of soil in the corresponding stages have not been clearly explored. In this paper, the alterations in the morphology of calcium carbonate crystals were continuously observed via scanning electron microscopy during the MICP process in one week, and the mechanical changes of the samples were monitored every day, so as to reveal the relationship between the morphology of calcium carbonate crystals and the mechanical performance of the samples. The results show that the calcium carbonate crystals undergo a gradual change from ellipsoid to rhombic at the 72nd hour. The mechanical properties of both were greatly improved, among which the compressive strength was increased by 2.78 times compared with the previous time point, and the flexure strength was increased by 2.57 times; this time point was also the time when calcite appears. In addition, we found direct evidence on the first day that bacteria act as the nucleation site of calcium carbonate formation. The above findings have certain guiding significance for the in-depth understanding of the internal microscopic changes of MICP and the influence of calcium carbonate morphology on sample mechanics.

## 1. Introduction

Microbially induced carbonate precipitation (MICP) is a technology that adheres to the surrounding sand or soil through the calcium carbonate produced by the life activities of microorganisms, thereby changing the mechanical properties of the surrounding soil [1,2]. The most common approach to MICP reaction is to utilize calcium carbonate induced by urea decomposition reaction initiated by urea hydrolyzing bacteria. The basic principle of MICP is that bacteria decompose urea to produce ammonium (NH_3_) and carbonate ions (CO_3_^2−^); ammonium reacts with water to form ammonium ions (NH_4_^+^) and hydroxide ions (OH^−^), thus improving the pH to create an alkaline environment until NH_4_^+^ /NH_3_ and HCO_3_^−^/ CO_3_^2−^, reaching equilibrium [3]. Under an alkaline environment, Ca_2_^+^ and CO_3_^2−^ react to generate CaCO_3_ precipitation [4]. The reaction process can be expressed by Equations (1) and (2):

CO(NH_2_)_2_ + 2H_2_O→CO_3_^2−^ + 2NH_4_^+^ (1)

Ca^2+^ + CO_3_^2−^→CaCO_3_↓ (2)

Calcium carbonate induced by bacteria during the MICP process plays a central role in gluing loose soil or sand particles together [5,6]. Through the adhesion of calcium carbonate, the permeability of the soil can be significantly reduced and the mechanical strength of the soil can be improved. The main existing forms of calcium carbonate are spindle, cube, needle, flake, and rod, etc. [7,8]. Due to differences in shape, its properties are not the same [9,10]. Therefore, it is significant to explore the development process of calcium carbonate crystals in the process of MICP, for us to understand the effect of different morphologies of calcium carbonate on the mechanics of soil treated with MICP. The MICP process is a complex process, during which the crystal form of calcium carbonate changes. As observed by Chu and Blanco, rhombohedrals are often seen in late MICP and spherical calcium carbonates appear in the mid-phase. Blanco proposed that rhombohedral calcium carbonate corresponds to calcite, and spherical calcium carbonate corresponds to vaterite based on the appearance and stability of different forms of calcium carbonate [11,12].

According to previous studies, bacteria act as the initial nucleation site for calcium carbonate [13,14,15,16], which then continues to grow in situ. This hypothesis is based on the fact that the bacterial cell wall can adsorb cations to combine with carbonate to generate calcium carbonate [17]; however, there is no direct picture evidence that bacteria are encapsulated by calcium carbonate as a nucleation site, so this argument has been questioned by some scholars. Some scholars have also proposed the enzyme-induced carbonate precipitation (EICP) theory [18], arguing that the existence of bacteria is not necessary for the mineralization process, as long as there is an enzyme that hydrolyzes urea [19,20]. Wang et al. used a microfluidic platform to explore the nucleation and growth changes of calcium carbonate in the early stage of MICP, and also explored the effect of bacterial concentration on the growth of calcium carbonate crystals [7]. The results showed that irregularly shaped CaCO_3_ precipitates initially emerged on bacterial aggregates and subsequently dissolved with time as regularly shaped CaCO_3_ crystals started growing. Gowthaman et al. conducted a detailed and in-depth study of the mechanical behavior of soil after MICP treatment, such as freeze–thaw (FT) response and shear stress [21,22]. However, no study has yet combined the change of calcium carbonate morphology during the MICP process with the corresponding mechanical performance to explore the effect of calcium carbonate morphology on soil samples. In addition, the observation of morphological changes on MICP was not extended to a longer time period (more than 24 h), so it is still unclear how calcium carbonate transforms from the irregular form to the calcite form in the late stage.

In this study, two different sizes of molds were designed for the observation of microscopic calcium carbonate crystals and the mechanical tests of the samples. Scanning electron microscopy (SEM) was utilized to explore the changes in the morphology of calcium carbonate during the MICP process (7 days). At the same time, the unconfined compressive strength test and three-point bending experiments were conducted to monitor the daily mechanical alterations of the samples to find the mechanical relationship corresponding to the crystal morphology. Lastly, acid hydrolysis tests and water absorption tests were used to reflect the calcium carbonate content of each sample and the density of calcium carbonate.

## 2. Materials and Methods

### 2.1. Materials

#### 2.1.1. Bacterial Cultivation

In this study, *Sporosarcina pasteurii* stored in our laboratory was utilized to conduct the experiments. The activation and cultivation of the bacteria was conducted via the medium, including 20 g/L yeast extract, 10 g/L ammonium sulfate, and 2 g/L Tris. The pH of the medium was pre-adjusted to 8.0, then the bacterial culture medium was placed in a constant temperature shaker at 28 °C and incubated at 200 rpm until the OD_600_ value was 3.45 ± 0.3, and the urease activity was 15.12 ± 0.2 mM/min [23].

#### 2.1.2. Sand

**Figure 1:**
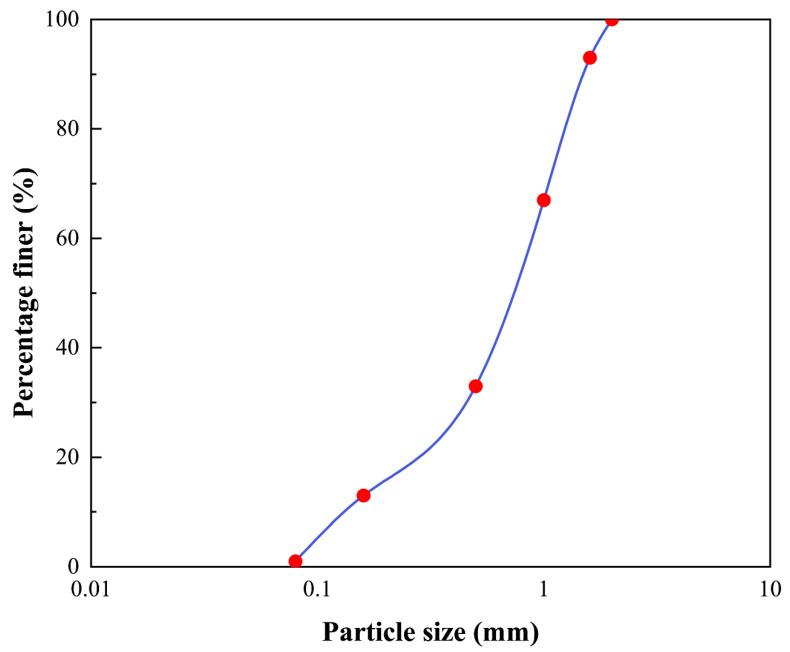
Particle size distribution of the sand.

Sand with a specific gravity of 2.65, a coefficient of uniformity (C_u_) of 6.77, and a coefficient of curvature (C_c_) of 1.77 were utilized in this study. The sand particle distribution is shown in Figure 1. All sand grains were washed three times using double distilled water in preparation to remove impurities. Then, the sand is naturally dried and used for subsequent sample preparation.

#### 2.1.3. Specimen Preparation

**Figure 2:**
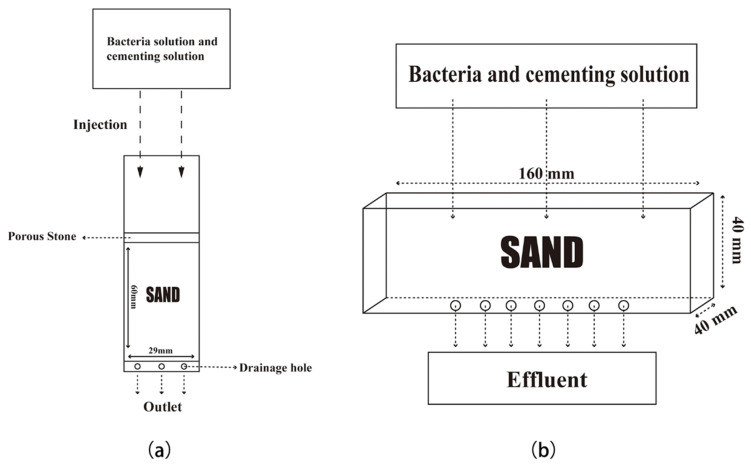
Two molds used in this research: (**a**) 29 × 60 mm size mold; (**b**) 40 × 40 × 160 mm size mold.

Two different molds filled with sand were set up for different experimental purposes. The specimen with the diameter of 29 mm and height of 60 mm was prepared by the plastic cylinder referred to by Choi et al. [16], shown in Figure 2a. Then, 78 ± 1 g of sand was loaded into the mold in three batches; each filling was compacted with a plastic plate and the sand was pressed to the height of around 20 mm each time. Through the water saturation weighting procedure [16], we knew that its pore volume was about 10 mL. Before MICP treatment, it was filtered and washed with excess double-distilled water. After standing at room temperature for 24 h, 10 mL of bacterial liquid was injected into the mold, and then allowed to stay for 8 h to promote the adsorption of bacteria on the sand particles. Subsequently, 10 mL of the cementing solution (composed of 1M urea and 1M CaCl2 and pH was adjusted to 8 by 1 g/L of tris-based solution) was injected, and then the samples were placed under a temperature of 30 °C. After MICP processing, samples in this mold were taken out for SEM observation at various time periods.

In addition, a plastic cuboid model at a size of 40 × 40 × 160 mm was utilized to fill with sand for mechanical monitoring and permeability testing [14], as shown in Figure 2b. The pore volume of 70 ± 0.5 mL for sands filled in this mold was determined by the water saturation-weight method. Then, 460 ± 0.01 g sands were loaded into the mold for subsequent MICP treatment. Similar to the above treatment, 70 mL of bacterial liquid was injected into the mold, and the cementing liquid (composed of 1M urea and 1M CaCl_2_ and pH was adjusted to 8 by 1 g/L of tris-based solution) was injected after 8 h. Then, all the specimens were placed under 30 °C and taken for mechanical tests, calcite content tests, and permeability tests at different time periods.

### 2.2. Properties Tests

#### 2.2.1. Microscale Observation and Spectroscopy Analysis

The changes in calcium carbonate crystals during MICP were observed by scanning electron microscopy (FE-SEM, Sirion 200, FEI, Eindhoven, Netherlands). At the corresponding time point, the sand was taken out of the 29 × 60 mm mold and observed using a scanning electron microscope. There were three parallel samples in each time period to ensure the universality of the obtained images. The minerals were identified by X-ray powder diffraction spectrometry (XRD) analyses (XRD-7000 X-ray diffractometer, Shimadzu, Kyoto, Japan) with the PDF-2 database of the International Center for Diffraction Data.

#### 2.2.2. Unconfined Compressive Strength and Flexure Strength Test

The sand samples were taken from 40 × 40 × 160 mm molds to conduct the mechanical performance test. Unconfined compressive strength (UCS) and flexure strength of sand columns were tested using a Zwicki-line machine (Ulm, Germany) with 1mm/min loading speed in accordance with EN 196 [24,25].

#### 2.2.3. Water Absorption Test

The water absorption capacity of the specimen reflected inner and surface pore structure; it also revealed the number of pores. Referring to Manzur’s method [26], water absorption capacity was tested by the following procedures: First, place the sand column in a 100 °C oven for 24 h to remove all moisture, and record the mass m_1_ at this time. Then, put the dry sand column into the distilled water and submerge it below the water surface for 24 h. Mass after water absorption is recorded as *m*_2_. The water absorption of specimen *w* is calculated according to the Equation (3):

(3) $$w=\frac{m_{2}-m_{1}}{m_{1}}\times 100\%$$

#### 2.2.4. Calcite Content Tests

Calcite content was defined as the ratio of the calcite mass and the dry mass of the specimen, as shown in Equation (4):

(4) $$\mathrm{Calcite}\;\mathrm{Content}\;=\frac{m_{CaCO_{3}}}{m_{specimen}}\times 100\%$$

The sand columns were taken from the mold and the initial mass $m_{specimen}$ was recorded. Then, the sand columns were set in excess dilute hydrochloric acid to fully react for 24 h. When no more bubbles were generated in the reaction system, the sand was taken out and dried to remove excess water. The difference between the dried sand and the initial mass was the mass of calcium carbonate produced ($m_{CaCO_{3}}$) [27]. At this point, the calcium carbonate content was calculated using Equation (4).

### 2.3. Statistical Analysis

The results were expressed as the mean ± SD of the three experiments. Statistical analysis was performed by the Student’s *t*-test. A statistical significance was accepted at *p* < 0.05.

## 3. Results and Discussions

### 3.1. Morphology Alteration Process of Calcium Carbonate Crystals

**Figure 3:**
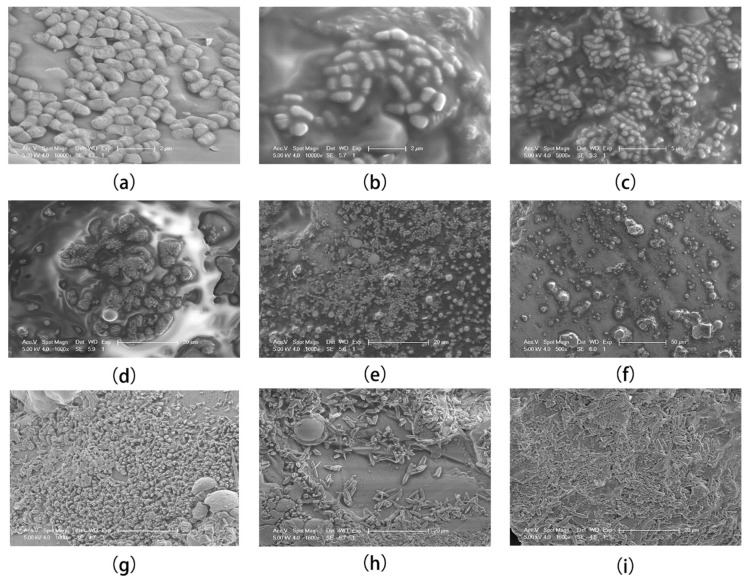
Morphology alterations of calcium carbonate crystals: (**a**) 2 h; (**b**) 4 h; (**c**) 6 h; (**d**) 12 h; (**e**) 24 h; (**f**) 48 h; (**g**) 72 h; (**h**) 120 h; and (**i**) 168 h.

After MICP treatment, sand column samples at different time points were collected and placed under SEM for observation. The first observation was made within two hours after the MICP reaction, at which time we found ellipsoids with similar distribution characteristics of bacteria, each with an average diameter of 2 μm and a size similar to the average length of bacteria (Figure 3a–c). This strongly supported the hypothesis of bacteria as calcium carbonate nucleation sites; at the 4 h, we then observed that the calcium carbonate spheroids were gradually glued together (Figure 3b), the boundaries between the spheroids became blurred, and the overall shape presented an irregular lump shape. In the interval of 6–48 h, the number of irregular lumps wrapped around calcium carbonate ellipsoids gradually increased (Figure 3d–f), and irregular calcium carbonate protrusions with small sizes sporadically appeared at the 24th hour (Figure 3e), and were more obvious at the 48th hour (Figure 3f). Notably, unidentified gelatinous substances have also been reported in the literature and were considered to be bacterial secretions [14]. The organic substances from bacteria coated on the surface of sand grains acted as a ‘‘glue’’ to bond sand grains with biogenetic calcite via intermolecular hydrogen bonds [28].

At 72 h, a sudden and obvious change in the morphology of calcium carbonate crystals was observed: the morphology of calcium carbonate crystals changed from round to sharp and were distributed in clusters, similar to the morphology of rhombohedral calcite (Figure 3g). However, the rhombus was not obvious at this time, and the size was very small. As time progressed, the rhombus became more and more obvious. At the 120th hour, slender rhombus crystals were observed until the seventh day (168 h), which showed an obvious shape of rhombic calcite (Figure 3h,i). This form was also a stable calcium carbonate form, often observed in the late stage of MICP. According to the literature, the spherical and rhombohedral shapes of CaCO_3_ crystals are consistent with those of vaterite and calcite, respectively [12,29]. Therefore, during the MICP process, calcium carbonate mainly undergoes a transformation from vaterite to calcite, in which calcite exists as a stable calcium carbonate form, and the most important contributor to the improvement of soil mechanics. At the initial stage of the MICP reaction (Figure 3a–c), the morphology of the calcium carbonate-coated bacteria was paid more attention, so a smaller scale was chosen. In the later stage of MICP, a larger scale was chosen to demonstrate the generality of the calcium carbonate morphology transition (Figure 3d–i).

In general, during the MICP process, the bacteria were first coated with calcium carbonate, then adhered by unknown colloidal substances to form irregular shapes, and finally turned into rhombohedral calcite distributed in clusters. The calcium carbonate formed in the early stages of MICP has been considered to be an unstable state, and many factors affect its morphology and distribution, such as grouting intervals, bacterial concentration, and temperature [30]. In this study, only one grouting treatment was carried out, and focus was given to the observation of its morphology development; the influence of other factors on the morphology was avoided as much as possible. However, the process of the conversion of unstable calcium carbonate to rhombic calcite is still unclear, and what induces irregular calcium carbonate to calcite morphology requires further experimental exploration.

**Figure 4:**
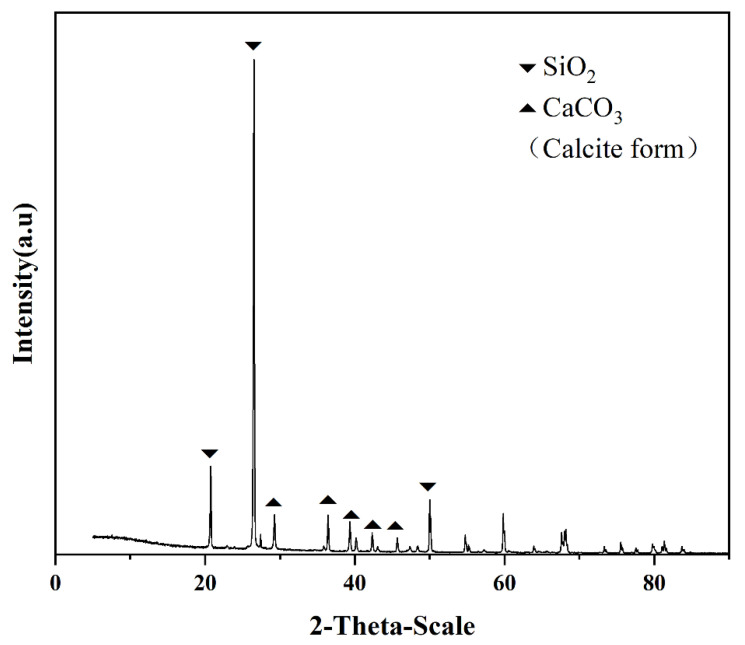
XRD pattern of sand columns after MICP process.

The XRD patterns of all specimens showed the calcite phase of calcium carbonate, with diffraction peaks at 2*θ* = 29.4°, 36.0°, 39.4°, 43.1°, 47.5°, and 48.5°, which refer to its (104), (110), (113), (202), (018), and (116) facets, respectively (JCPDS PDF2 standard card 05–0586), and confirmed that the crystals formed were calcite (Figure 4).

### 3.2. Mechanical Performance of Sand Columns

**Figure 5:**
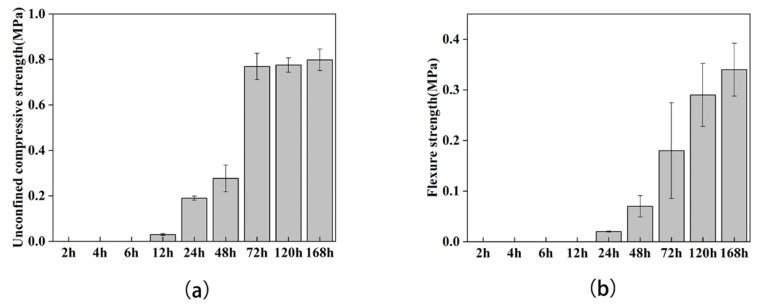
Mechanical performances of sand columns at different time point. (**a**) Unconfined compressive strength of sand columns; (**b**) flexure of sand columns.

The samples (40 × 40 × 160 mm) at each observation time point were selected to conduct mechanical experiments to explore the effects on the mechanical properties of the samples during the formation and development of calcium carbonate. The results showed that the flexural and compressive strengths of the samples increased with time and both reached their peaks on the seventh day. In the first six hours, the sand columns were still in a state of loose sand, and the corresponding mechanical performances could not be tested. The compressive strength was obtained at the 12th hour; however, the flexural strength was obtained at the 24th hour before the toughness was too low to be detected by the machine. We found that at 72 h, the mechanical properties of both were greatly improved, among which the compressive strength was increased by 2.78 times compared with the previous time point, and the flexure strength was increased by 2.57 times (Figure 5a,b). It is worth noting that this time period was the node where rhombohedral calcite appeared. In general, the flexural strength showed a step-by-step increasing trend, while the increase in compressive strength tended to moderate after the 72nd hour. The flexural strength varied greatly among the samples, while the compressive strength varied less among the samples.

The compressive strength is related to the porosity of the sample, and there is a basic inverse relationship between the two. The compressive strength increases with the decrease of the porosity, but this relationship may be linear or exponential [31,32]. The porosity of the soil samples treated by MICP decreases with the increase of curing time, and the decrease of water absorption is the manifestation of the decrease of porosity. When the porosity decreases to a certain level, the compressive strength of the sample significantly increases. We observed that the time node of the sudden increase of the compressive strength of samples occurred from 48 h to 72 h, when the water absorption significantly decreased, and then, with the stabilization of the porosity, its compressive strength also tended to be stable.

As for flexural strength, it mainly depends on the static friction between the calcium carbonate and the sand particles (cohesive force of the matrix) [33]. If the soil particles are not broken, only the calcium carbonate that plays a role in adhesion has an effect on the flexural strength [34]. Since the growth of calcium carbonate does not have a sudden change process, the growth of flexural strength is more continuous than that of flexural strength.

### 3.3. Water Absorption and Calcite Content Test

**Figure 6:**
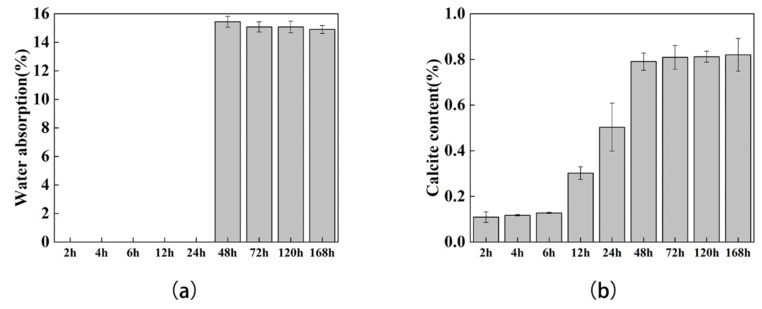
Water absorption and calcite mass test: (**a**) water absorption test results of specimens, (**b**) calcite content test results of specimens.

According to previous literature reports, the formation of calcium carbonates will reduce the permeability of soil [35], and the decrease in permeability reflects the generation and distribution of calcium carbonate to a certain extent. Here, the permeability of sand columns at different time points was tested; the sand columns in the first 24 h cannot be taken out after being soaked in the water, so they were regarded as invalid data. The results showed that with the extension of days and the increase of calcium carbonate content, the permeability gradually decreased (Figure 6a), but the amplitude was not obvious. Correspondingly, we observed that the content of calcium carbonate increased every day (Figure 6b), and the most significant increases were from 12 h to 24 h and 24 h to 48 h, which were 1.67 times and 1.57 times, respectively. 

Compared with the most significant increase in mechanical performance from 48h to 72 h, the significant increase period of calcium carbonate content was 24 h earlier. We speculated that a stable period was required after the formation of calcium carbonates, and that only the calcium carbonate in the stable state could act as a practical contributor to the enhancement of soil mechanical properties. This hypothesis may explain why the mechanical properties of the early sand columns were very poor, when the calcium carbonate was still in an unstable state. In addition, despite a slight increase in calcium carbonate content from 48 h to 168 h, considering the existence of sample differences, we are more inclined to believe that the MICP reaction stopped at 48 h, and no new calcium carbonate was generated from that point. This might be the reason why many MICP applications require multiple replenishments of bacterial and cementing liquids in order to generate more new calcium carbonates.

## 4. Conclusions

In this experiment, two different sizes of molds were used to explore the development trend of calcium carbonate crystal morphology and the corresponding mechanical and permeability performance during the MICP process. The main conclusions were as follows:

(1) Bacteria are thought to play a central role in the MICP reaction as calcium carbonate nucleation sites; the calcium carbonate-encapsulated colonies within two hours of the start of MICP were captured, demonstrating that this is direct evidence for bacteria as calcium carbonate nucleation sites.
(2) During the MICP process (7 days), the morphology alterations of calcium carbonate underwent the following processes: For the first two hours, the bacteria were encapsulated by calcium carbonate as nucleation sites. At this time, the calcium carbonate was an ellipsoid with a length of about 2 μm. From 4 h to 48 h, the ellipsoid calcium carbonate was wrapped by unknown colloids to form irregular lumps with protrusions. Notably, unidentified gelatinous substances have also been reported in the literature and have been considered to be bacterial secretions. From 72 h to 168 h, the calcium carbonate crystals changed from round to sharp, and they were densely distributed in clusters.
  This experiment only observed the change in trend of calcium carbonate morphology within 7 days, and did not follow up reports. In the next experiment, we will conduct experiments on calcium carbonate crystals in the long-term (more than 7 days) MICP reaction to explore further.
(3) The mechanical experiments showed that, with the increase in calcium carbonate production, the corresponding flexural and compressive strengths also increased, which was consistent with the phenomenon reported in the literature. At the same time, with the increase of calcium carbonate, the permeability of the sand column also decreased, which indicates that calcium carbonate reduced the porosity of the soil.

The morphology of calcium carbonate in different periods and the corresponding mechanical properties were linked together for the first time, which explains the influence of the difference of calcium carbonate crystals on the flexural and compressive properties of soil to a certain extent. The discovery of the relationship between calcium carbonate crystal morphology and mechanics provides theoretical support for subsequent civil engineering applications of MICP. Meanwhile, bacteria coated with calcium carbonate were observed in the early stages of MICP reaction, which strongly supports the hypothesis that bacteria act as calcium carbonate nucleation sites.

Although this study observed and recorded the growth changes of calcium carbonate in the process of MICP over a long period of time, there are still some details that are not clear, such as the unidentified gelatinous substance that wraps calcium carbonate cells in the early stages of MICP. In addition, the difference between the mechanical changes of flexural and compressive resistance is worth further exploration. Furthermore, the emission of by-product ammonia in MICP treatment presents a certain challenge to the large-scale application of MICP. Ammonia emissions could be reduced by urease inhibitor or zeolite treatment [36]. Whether the treatment processes above will interfere with the formation and development of calcium carbonate, further relevant experiments are highly recommended.

## Data Availability

The datasets generated during and/or analyzed during the current study are available from the corresponding author on reasonable request.
